# High-Performance Polyamide Reverse Osmosis Membrane Containing Flexible Aliphatic Ring for Water Purification

**DOI:** 10.3390/polym15040944

**Published:** 2023-02-14

**Authors:** Chi Jiang, Zhaohui Fei, Yingfei Hou

**Affiliations:** State Key Laboratory of Heavy Oil Processing, College of Chemistry and Chemical Engineering, China University of Petroleum (East China), Qingdao 266580, China

**Keywords:** reverse osmosis, interfacial polymerization, molecular structure, high permeance

## Abstract

A reverse osmosis (RO) membrane with a high water permeance and salt rejection is needed to reduce the energy requirement for desalination and water treatment. However, improving water permeance while maintaining a high rejection of the polyamide RO membrane remains a great challenge. Herein, we report a rigid–flexible coupling strategy to prepare a high-performance RO membrane through introducing monoamine with a flexible aliphatic ring (i.e., piperidine (PPR)) into the interfacial polymerization (IP) system of trimesoyl chloride (TMC) and m-phenylenediamine (MPD). The resulted polyamide film consists of a robust aromatic skeleton and soft aliphatic-ring side chain, where the aliphatic ring optimizes the microstructure of polyamide network at a molecular level. The obtained membranes thereby showed an enhanced water permeance of up to 2.96 L·m^−2^ h^−1^ bar^−1^, nearly a 3-fold enhancement compared to the control group, meanwhile exhibiting an ultrahigh rejection toward NaCl (99.4%), thus successfully overcoming the permeability–selectivity trade-off limit. Furthermore, the mechanism of the enhanced performance was investigated by molecular simulation. Our work provides a simple way to fabricate advanced RO membranes with outstanding performance.

## 1. Introduction

The shortage of freshwater is posing threats to the sustained development of both developed and developing countries [1,2]. Membrane filtration technologies, particularly reverse osmosis (RO), are extensively used to purify water (remove dissolved salts from water) [3]; nonetheless, the energy requirement remains high [4,5]. Numerical calculation studies have confirmed that the operation pressure and energy consumption applied to seawater RO and brackish RO can be decreased significantly when using an RO membrane with an enhanced water permeance and salt rejection [6,7]. Unfortunately, the improvement in membrane water permeance is generally accompanied by a compromised salt rejection due to the trade-off limitation of permeability and selectivity [8,9].

The state-of-the-art RO membrane isa thin-film composite (TFC) membrane containing an ultrathin dense separation layer and a porous substrate [10]. The separation layer, which mainly determines the separation performance, is synthesized by interfacial polymerization (IP) between amine dissolved in water and acyl chloride dissolved in an organic solvent [11]. During the IP process, amine molecules diffuse into the organic phase, and then react with trimesoyl chloride molecules [12]. Such a diffusion–reaction process results in a cross-linked polyamide film covering on the substrate. It is generally recognized that the polyamide film has two kinds of pores, i.e., network pores (free volumes within molecular chains) and aggregate pores (interspaces between the aggregates) [13]. These sub-nanometer pores provide a diffusion path for water molecules during the RO separation process. To this date, the combination of m-phenylenediamine (MPD) and trimesoyl chloride (TMC) is most commonly used for fabricating RO membranes. This MPD-TMC chemistry (well known as FT-30 chemistry [14]) is the basis of most commercial TFC RO membranes, although various novel materials have been studied over the past few decades.

Obtaining fresh water from seawater and wastewater relies on the selective permeation of water through the polyamide layer. However, water transport is heavily restricted by the polyamide chains in a tortuous manner [15]; one of the reasons for this is the efficient packing of molecular chains during the IP process. The rigid plane structure of benzene rings within MPD/TMC and their interaction (e.g., π-π stacking) leads to a dense polyamide network, which is short of sufficient interconnected free volumes. Thus, extensive effort has been dedicated to creating more water transport channels in the polyamide layer to improve the membrane performance. One commonly used strategy to tailor the microstructural properties of the active layer is to incorporate nanomaterials (e.g., zeolite, graphene oxide, carbon nanotubes, and metal organic frameworks) into the layer [16,17,18,19,20]. Although more water transport channels can be obtained by creating interfacial voids or introducing the intrinsic voids of nanomaterials, the low affinity with the polymer matrix usually facilitates the formation of unselective defects, causing decreased salt rejection [21]. Most recently, Culp et al. revealed the relationship between the nanoscale polyamide structure and membrane performance and demonstrated that significance of controlling nanoscale polyamide homogeneity (increasing uniformly distributed angstrom-scale free volume polyamide) [22]. However, due to the rapid and uncontrolled reaction at the nanometer–thick interface region, achieving the desired polyamide properties with optimized microstructures remains a scientific and technological challenge.

**Figure 1:**
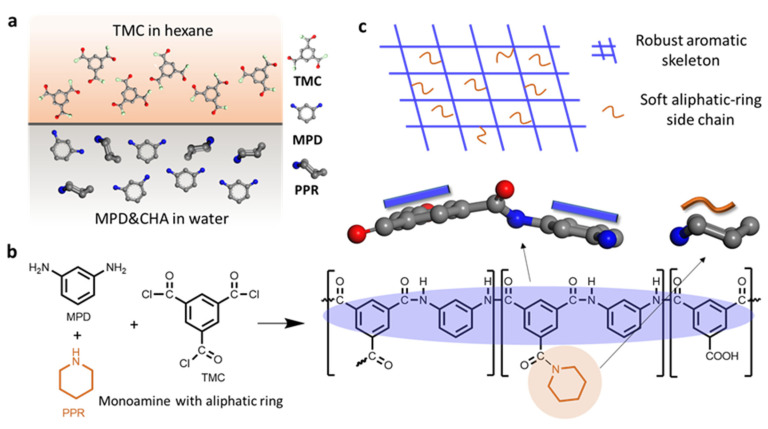
Illustration of the rigid–flexible coupling strategy for the fabrication of a high-performance RO membrane. (**a**) Visualization of interfacial polymerization between TMC and MPD and PPR; (**b**) synthesis of the polyamide consisting of a robust aromatic polyamide skeleton and a soft aliphatic-ring side chain; (**c**) schematic diagram of the rigid–flexible coupling polyamide network.

In this work, the RO membrane with an enhanced water permeance and salt rejection was prepared by designing the polyamide structure at the molecular level. As shown in Figure 1, a monoamine with aliphatic ring (e.g., piperidine (PPR)) was introduced into the conventional IP system of TMC and MPD to tailor the intrinsic properties of polyamide layer. Different from insoluble nanomaterials, monoamine uniformly dissolves in the aqueous phase and then diffuses into the organic phase to react with TMC (Figure 1a). Due to the self-limitation of single reactive groups, the participation of monoamine will not replace MPD in a cross-linked polyamide. The resulting polymer consists of a robust aromatic polyamide skeleton and soft aliphatic-ring side chain (Figure 1b). Due to the regulation of aliphatic ring on microstructure of the polyamide network (Figure 1c), the obtained RO membrane showed an enhanced water permeance and NaCl rejection. In this work, the fabricated RO membrane containing robust aromatic ring and soft aliphatic ring is named a rigid–flexible coupling RO (RFRO) membrane.

## 2. Materials and Methods

### 2.1. Materials

The polymer substrate used to prepare the TFC RO membranes was a PSF polysulfone (PSF) ultrafiltration membrane, which was purchased from Beijing OriginWater Membrane Technology Co., Ltd. (Beijing, China). The commonly used monomer to build the polyamide separation layer, i.e., m-phenylenediamine (MPD, 99%), and trimesoyl chloride (TMC, 98%), were purchased from TCI Chemicals (Tokyo, Japan). The additives to prepare RFRO membranes, i.e., PPR, and the additives used in the control group, i.e., piperazine (PIP) were purchased from Aladdin (Shanghai, China). The solute to test the membrane performance, i.e., sodium chloride (NaCl, 99.5%), was purchased from Sinopharm Chemical Reagent Co., Ltd. (Shanghai, China). Deionized (DI) water produced by a two-stage RO system was used throughout all experiments.

### 2.2. Preparation of RO Membranes

The TFC RO membranes were prepared by the IP method. For the fabrication of the conventional RO membrane as the control, the PSF ultrafiltration substrate was immersed in a 2 *w*/*w*% MPD aqueous solution for 5 min, and the excess MPD solution was removed with an air gun until no water droplet was observed. Then, the 0.1 *w*/*w*% TMC dissolved in n-hexane solution was carefully poured onto the substrate saturated by aqueous phase to generate the IP reaction and was held for 30 s. Subsequently, the resulting membrane was washed with pure n-hexane. Finally, the obtained membrane was placed in an oven at 60 °C for 2 min to form a stable cross-linked structure. For the fabrication of RFRO membranes, a certain amount of monoamine with an aliphatic ring (i.e., PPR) was added into the MPD aqueous solution, and the resulting mixed solution was used as an aqueous phase for IP. The other steps and conditions were the same as the fabrication of a conventional RO membrane. In the following discussion, the PPR concentration was the mass concentration of PPR in the aqueous phase.

### 2.3. Characterization

The chemical compositions of the substrate and RO membranes were characterized by attenuated total reflectance-Fourier transform infrared (ATR-FTIR) spectroscopy (NICOLETTM iS10 spectrometer, Thermo Fisher Scientific, Waltham, MA, USA) and X-ray photoelectron spectroscopy (XPS, Thermo Scientific K-Alpha, USA) tests. The surface morphologies were measured by a scanning electron microscope (SEM, TESCAN MIRA LMS, CZ, Brno, Czech Republic) and an atom force microscope (AFM, Shimadzu SPM-9700, Kyoto, Japan) with contact model, and the surface roughness was evaluated by the parameter of room mean surface roughness (R_q_). The cross-sectional structural properties and internal nano-sized voids of the RO membranes were charactered by transmission electron microscopy (TEM, JEM 1200EX, JEOL, Tokyo, Japan). The TEM test samples were prepared by ultra-thin-sectioning according to the following procedures: the RO membrane was cut into a strip shape, and then immersed in a series of water/ethanol solutions to dehydrate the membrane. Subsequently, the dried membrane was embedded in LR resin and held for curing. Finally, the embedded membrane was sliced into ultrathin sections (this thickness was about 80 nm) to obtain the sample for TEM measurement.

### 2.4. Performance Evaluation

The separation performance of the fabricated membranes was evaluated by water permeance, NaCl rejection rate (2000 mg/L NaCl solution), and long-time stability. The desalination tests were carried out based on a laboratory-scale cross-flow apparatus with six filtration cells (each cell had an effective area of 17.7 cm^2^). The tests were carried out with an operation pressure of 15 bar, a feed flow rate of 4.5 L min^−1^, and a test temperature of 25 °C. Each membrane sample was pre-pressed for at least 2 h at 15 bar to reach a stable state. The water permeance and salt rejection rate were calculated according to Equations (1) and (2), respectively.

(1) P =V/(A × t × P)

(2) $$R\left(\%\right)=\left(1\;-\;\frac{C_{p}}{C_{f}}\right)\;\times \;100\%$$

where *P* is the water permeance (L m^−2^ h^−1^ bar^−1^), *V* is the volume of the permeated water (L) collected within time t (h), A is the effective membrane area (m^2^), and C_p_ and C_f_ represent the salt concentrations of the permeated solution and feed solution, respectively (obtained by a conductivity meter).

### 2.5. Molecular Simulation

To obtain the information about the microstructure of the membrane materials, a molecular dynamic (MD) simulation was applied to build the molecular models of the cross-linked polyamide and to analyze their microscope properties including free volume and chain movability. Briefly, 150 TMC, 175 MPD, and 20 PPR molecules were blended, then the crosslinking simulation was carried out based on a script program in Materials Studio (during this process, a new amide group was created when the distance between acyl chloride in TMC and amine in MPD (or PPR) was below cut-off distance, until 70% of acyl chloride groups were consumed). Finally, the polyamide model was relaxed though an equilibration NPT MD simulation and NVT MD simulation to approach the real state of polyamide film [23]. The free volume properties of the models were analyzed by the Connolly surface method and the motion of the polymer chains was analyzed based on a root-mean-square displacement (MSD) curve of the polymer chain.

## 3. Results and Discussion

### 3.1. Chemical Composition and Structural Properties of the RO Membrane

**Figure 2:**
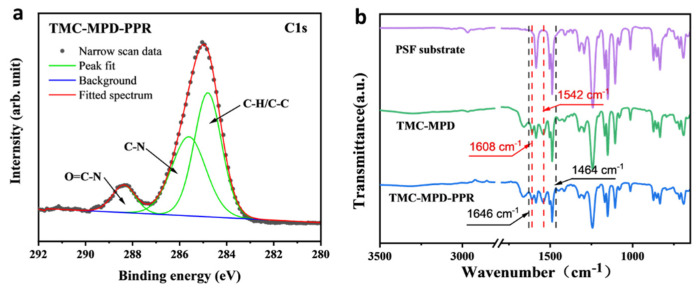
Chemical composition of the fabricated RO membranes. (**a**) C1s XPS spectra of the membranes; (**b**) ATR-FTIR spectra of the PSF support and prepared RO membranes; the concentration of PPR for modified RO membrane was 1.0%.

The chemical composition of the membranes fabricated by IP was analyzed by XPS characterization. Figure 2a shows the resolved C1s XPS spectrum of the fabricated RFRO membrane. Three peaks arising from O=C–N, C–N, and C–H/C–C bonds were observed, demonstrating the formation of polyamide. From the comparison of O1s XPS spectra (Figure S1), it was found that the carboxyl group content of the polyamide membrane was reduced when the PPR was added in the IP system (from 31% to 19%). This was because the PPR consumed a part of acyl chloride groups of TMC; thus, the unreacted acyl chloride groups that subsequently hydrolyzed into carboxyl groups decreased. IR characterization (Figure 2b) further showed the difference between the conventional RO membrane and RFRO membrane. Compared with the PSF substrate, the IR spectra of RO membrane showed two absorption peaks at 1608 cm^−1^ and 1542 cm^−1^, which corresponded to the stretching vibration of C=O and bending vibration of N-H in the full-aromatic polyamide groups formed by the polymerization of MPD and TMC. In addition to the above absorption peaks, two new absorption peaks at 1646 cm^−1^ and 1454 cm^−1^ were observed in the IR spectra of the RFRO membrane (TMC-MPD/PPR), which resulted from the stretching vibration of C=O in semi-aromatic polyamide formed by PPR and TMC. The above results demonstrate the fact the PPR and MPD co-reacted with TMC during the IP process, and formed the polyamide containing the benzene ring and aliphatic ring.

**Figure 3:**
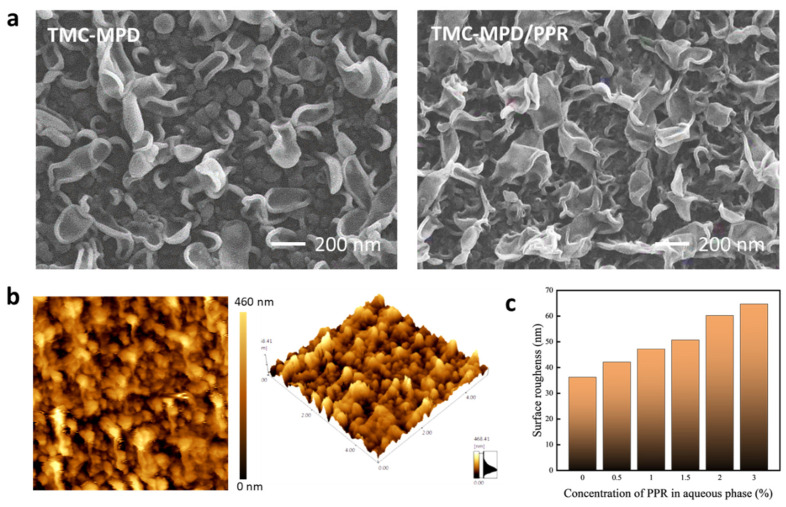
Structural properties of the fabricated RO membranes. (**a**) Surface SEM images of conventional RO membrane (TMC-MPD) and RFRO membrane (TMC-MPD/PPR; the concentration of PPR is 1.0%); (**b**) AFM height images of RFRO membrane; (**c**) variation of membrane surface roughness versus additional amount of PPR.

The structural properties of the fabricated membranes were characterized by SEM and AFM. Figure 3a shows the surface SEM images of the fabricated RO membranes. A typical “ridge-and-valley” morphology containing leaf-like structures and nodular structures was observed on the membrane surfaces, which may have originated from reaction heat [24], the release of gas bubbles [25], and the uneven loading of the amine monomer [26] during IP process. However, compared with the conventional RO membrane, the RFRO membrane showed more and larger leaf-like structures. These structures were expected to increase the membrane surface roughness, which was further proved by AFM measurement. As presented in Figure 3b, the surface roughness of the obtained membranes gradually increased from 36.2 nm to 64.7 nm with the addition of PPR into the aqueous phase for IP. The increase in surface roughness generally has a positive correlation with the effective filtration area, which favors the improvement of membrane permeance [27,28].

**Figure 4:**
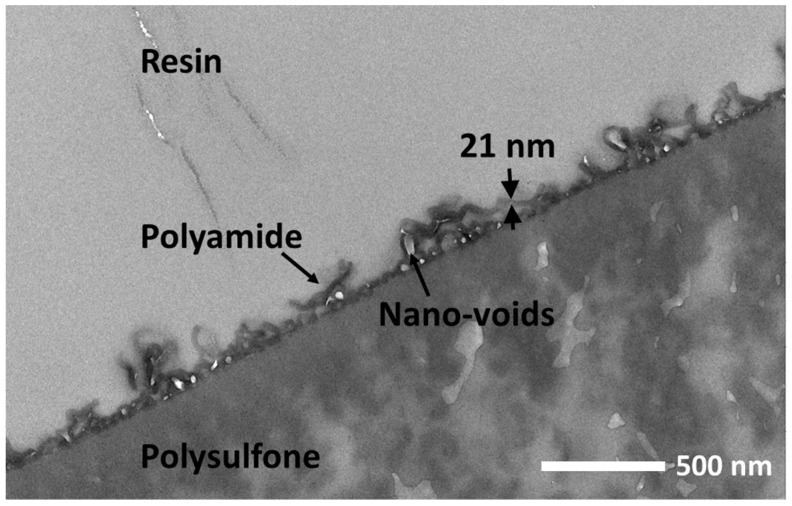
Cross-sectional TEM image of the RFRO membrane; the concentration of PPR is 1.0%.

Furthermore, the cross-sectional structure of the RFRO membrane was characterized by TEM measurements (Figure 4). The result shows the typical composite structure containing a continuous polyamide separation layer and a porous PSF substrate. The “ridge-and-valley” structures of polyamide layer were observed in the cross section, which confirmed the result from SEM measurement. Moreover, the TEM measurement also revealed the internal property of the rough polyamide separation layer. The abundant light grey regions demonstrate that the rough three-dimensional structures were hollow inside; these internal nano-voids are believed to serve as low-resistance pathways for water collected by the polyamide layer [29]. The TEM measurement also showed that the thickness of the polyamide film was about 21 nm. Such an ultrathin membrane thickness contributes to high water permeance because of the ultra-short distance for water permeation [30,31]. In addition, the TEM measurements for the TMC-MPD membrane were conducted to compare the variation in membrane thickness. As shown in Figure S2, the thickness of the polyamide layer was about 19 nm, which was slightly less than that of the membrane fabricated with PPR. We speculate that the participation of PPR decelerated the self-termination process of IP, thus leading to a slightly increased membrane thickness.

### 3.2. Separation Performance

**Figure 5:**
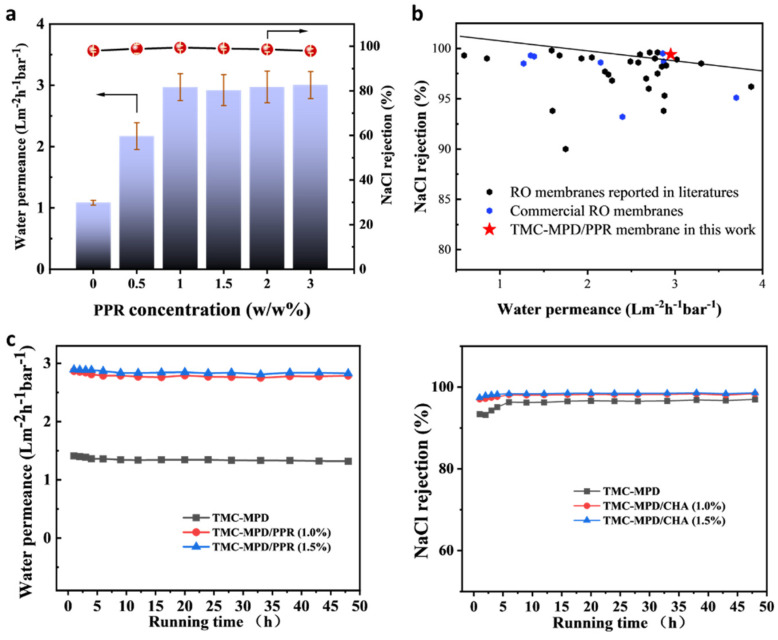
Performance of the fabricated RO membranes. (**a**) Variation of water performance and NaCl rejection of RFRO membranes versus the addition amount of PPR; (**b**) comparison of our fabricated RO membranes with commercial RO membranes and other advanced RO membranes reported recently; the line is the upper bound of performance, which was established based on the majority of the selected data; (**c**) operation stability test of the fabricated membranes.

The performance of RFRO membranes was evaluated by a cross-flow desalination test with a 2000 ppm NaCl solution as feed. Figure 4a shows the variation of water permeance and NaCl rejection versus the addition amount of PPR for fabricating RFRO membranes. Compared with the conventional RO membrane (the point of 0 PPR concentration in Figure 5a), a noticeable performance improvement was observed for the RFRO membrane prepared with the incorporation of PPR. The water permeance increased from 1.08 Lm^−2^ h^−1^ bar^−1^ to 2.96 Lm^−2^ h^−1^ bar^−1^ when 1% PPR was added, a near 3-fold improvement. Importantly, the rejection toward NaCl increased as well (from 98.1% to 99.4%). The simultaneous promotion in water permeance and salt rejection fully demonstrates the validity of the rigid–flexible coupling strategy for fabricating a high-performance RO membrane. However, when excess PPR was added (concentration above 1.5%), the separation performance of the obtained membrane would not continuously increase; this phenomenon may be attributed to the reaction self-limitation of monoamine during IP. Specifically, the reaction of PPR and TMC cannot form a polymer film due to the single reactive group of PPR; thus, it cannot replace the dominant role of MPD in cross-linked polyamide chains, even with an excess addition of PPR.

Figure 5b shows the comparison of our representative membranes and commercial RO membranes and other advanced RO membranes reported in the recent literature. Our membrane is located in the upper right corner of the figure, demonstrating its performance advantages both in water permeance and salt rejection in comparison with other RO membranes. To further evaluate the potential of the RFRO membranes in practical applications, the operational stability of the membranes was examined by a long-time cross-flow desalination test under an operation pressure of 1.5 MPa. As presented in Figure 5c, the water flux and NaCl rejection varied lightly for the first 5 h, and then remained stable for the following 45 h. In addition, the XPS measurement (Figure S3) shows that the chemical composition of the membrane remained unchanged after cross-flow filtration test. The above results demonstrate the RFRO membrane has great potential for a long-time operation. Consequently, it can be concluded that the rigid–flexible coupling strategy could be harnessed to fabricate advanced RO membranes.

### 3.3. Mechanism Analysis

**Figure 6:**
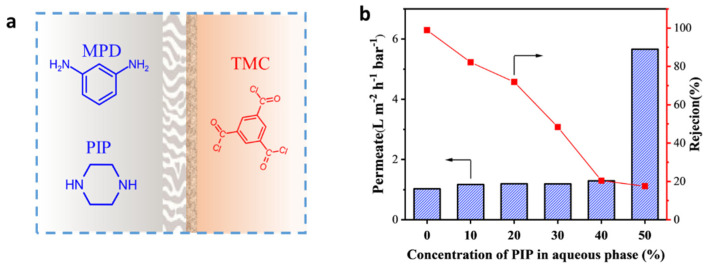
(**a**) Control groups for showing the key point for fabricating an RFRO membrane, where PIP (diamine with flexible aliphatic ring) was added into the aqueous phase for IP. (**b**) Performance of membranes fabricated by adding PIP.

The RFRO membranes with enhanced performance were fabricated by adding a monoamine with a flexible aliphatic ring (PPR) into the aqueous phase (MPD solution) for IP. The key point for our proposed rigid–flexible coupling strategy was the fact that the added second monomer only had one reactive amine group. To show the necessity of the above character of the additive, the control group was designed where a diamine with flexible aliphatic ring (i.e., PIP) was added into the aqueous phase to fabricate the RO membrane by the IP method (Figure 6a). Figure 6b shows the performance of the obtained membranes. A significant decrease in NaCl rejection was observed. Although we changed the addition amount, a membrane with an acceptable performance was not obtained. In addition, the SEM measurement (Figure S4) showed that the membrane fabricated by adding PIP had a surface morphology between a typical RO membrane and a typical NF membrane. Actually, PIP is the most commonly used amine monomer for fabricating a nanofiltration (NF) membrane, which generally has a higher water permeance than an RO membrane, but a much lower NaCl rejection (~30%) [32]. Although PIP has a similar flexible aliphatic ring with PPR, the introduction of PIP did not bring the hoped-for enhanced water permeance, but instead had a terrible performance in relation to rejection. We speculated that the completely different result caused by PIP was due to the following reason: PIP and MPD co-reacted with TMC during the IP process, which changed the skeleton structure of the cross-linked polyamide network, forming a membrane containing TMC/PIP micro-phase and TMC/MPD micro-phase regions. The TMC/PIP micro-phase was actually a “defect” region for the RO desalination process; thus, the resulted membrane exhibited an unsatisfactory performance.

**Figure 7:**
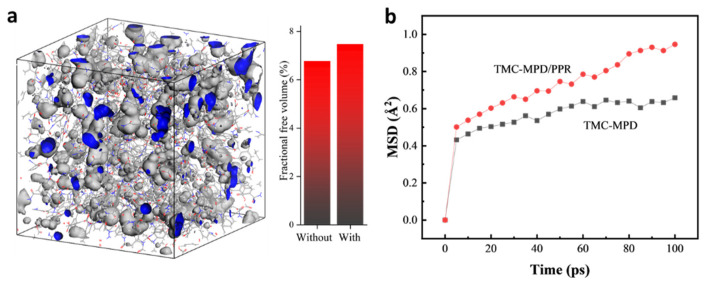
(**a**) Micro-properties of polyamide analyzed by molecular simulation; three-dimensional view of the free volume in the molecular model of polyamide synthesized by the reaction of TMC, MPD, and PPR; the probe radius is 1.4 Å; the outside surface of free volume region is shown in gray, and the interior of free volume is shown in blue; the right histogram shows the comparison of FFVs of polyamide models constructed with or without PPR; (**b**) mean square displacement (MSD) of polymer chains versus simulation time.

To further investigate the mechanism of the advanced performance offered by adding PPR, a molecular simulation was used to construct the molecular model of a rigid–flexible coupling polyamide and to reveal the micro-properties of the materials, including the free volume and motion of the polymer chains. Figure 7a shows the cross-linked molecular model of materials synthesized by TMC, MPD, and PPR, wherein the free volumes calculated with a probe with radius of 1.4 Å are visualized by blue and gray colors. Because the radius of a water molecule is about 1.4 Å, the observed free volumes were all accessible for water. For better comparison, the fractional free volumes (FFVs) of various materials were calculated and the results are presented in Figure 7a. The FFV value of TMC-MPD/PPR polyamide was larger than that of full-aromatic polyamide (TMC-MPD). It is believed that the free volumes serve as pathways for water diffusion in polyamide layer. Thus, the enhanced water permeance of RFRO membrane can be attributed to the increased free volumes, which originated from the regulation effect of the flexible aliphatic ring on the distribution of polyamide chains. In addition, we believe that the regulation effect from the flexible aliphatic ring also reduced the nanoscale defects. Thus, the obtained membrane showed an enhanced NaCl rejection rate.

According to the hopping mechanism which describes the diffusion of small molecules in polymers, the small molecules may oscillate in a particular void (free volume) and when a proper pathway is created by the polymer chains moving, it occasionally jumps into a neighboring void [33]. Thus, the motion of polyamide chain is an important factor influencing the diffusion of water in polyamide layer. The motion properties of the polyamide chains with or without PPR ring were investigated by a molecular simulation, where the slope of the MSD–time curve reflected the moveability of polymer chain. As presented in Figure 7b, the slope of TMC-MPD/PPR model was larger than that of TMC-MPD model, which demonstrates that moveability of polyamide chain was enhanced when introducing the flexible aliphatic ring. On the basis of the results of the experimental characterization and molecular simulation, it can be concluded that the enhanced separation performance of RFRO membrane is attributed to the optimized molecular structures (corresponding to enhanced diffusion of water molecules in polyamide layer) and increased the surface roughness (corresponding to increased filtration area).

## 4. Conclusions

In this work, we reported the fabrication of high-performance RO membrane through a rigid–flexible coupling molecular design strategy. A monoamine with flexible aliphatic ring was added into the TMC-MPD IP system, resulting in a polyamide film consisting of a robust aromatic skeleton and a soft aliphatic-ring side chain. The introduction of the soft aliphatic ring not only regulated the packing of the polymer network during the IP process to create more free volumes but also to increase the moveability of the polymer chains as demonstrated by the molecular simulation, which favored the diffusion of water molecules in the polyamide layer. In addition, the synthesized RO membrane also has an increased surface roughness, offering a more effective area for water permeation. The obtained RFRO membranes showed a simultaneously enhanced water permeance and salt rejection. This work provided a novel designing scheme for fabricating an RO membrane with an outstanding performance.

## Data Availability

Not applicable.

## Supplementary Materials

The following supporting information can be downloaded at: https://www.mdpi.com/article/10.3390/polym15040944/s1.
