# Supplementary material for: High-Performance Polyamide Reverse Osmosis Membrane Containing Flexible Aliphatic Ring for Water Purification
Source: Polymers (Basel). 2023 Feb 14;15(4):944. doi: 10.3390/polym15040944 (PMC9965555; doi:10.3390/polym15040944)
Supplement: Supplementary file 1 [file polymers-15-00944-s001.zip › polymers-2193539-supplementary.pdf]

Supplementary Material

High-performance polyamide reverse osmosis membrane containing flexible aliphatic ring for water purification

Chi Jiang*, Zhaohui Fei and Yingfei Hou*

State Key Laboratory of Heavy Oil Processing, College of Chemistry and Chemical Engineering, China University of Petroleum (East China), Qingdao 266580, China

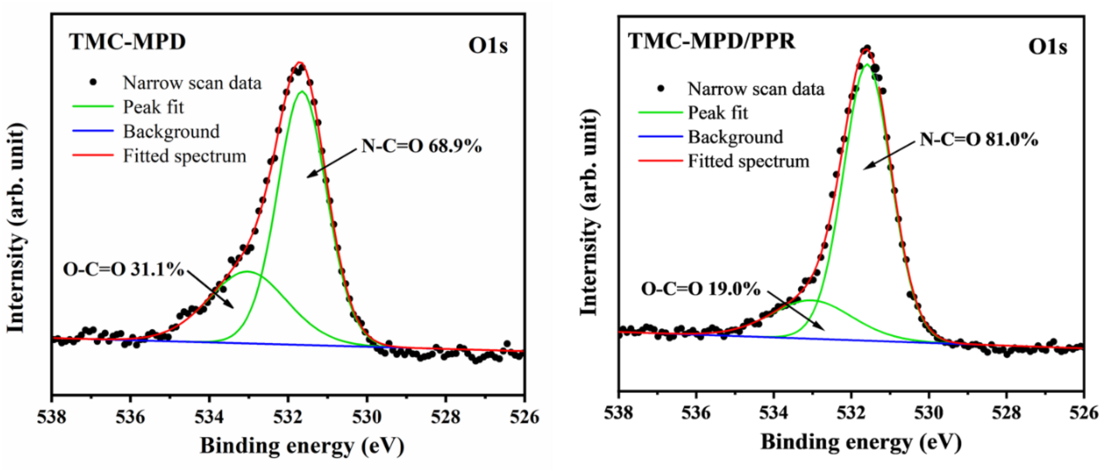

Figure S1 O1s XPS spectra of the pristine membrane (TMC-MPD) and the membrane fabricated by adding 1.0% PPR (TMC-MPD/PPR)

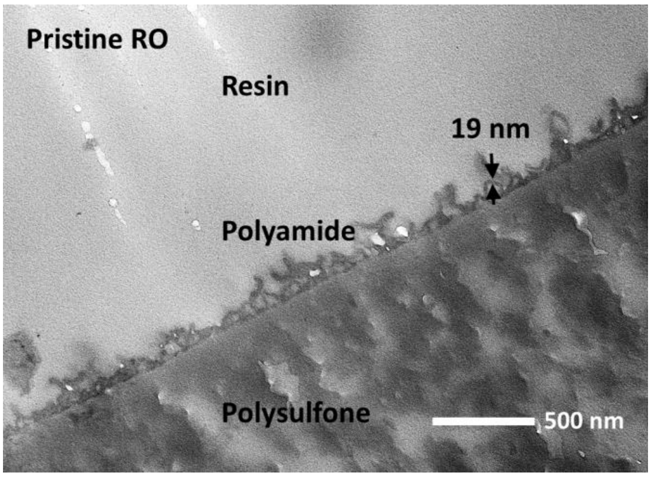

Figure s2 Cross-sectional TEM image of the pristine RO membrane

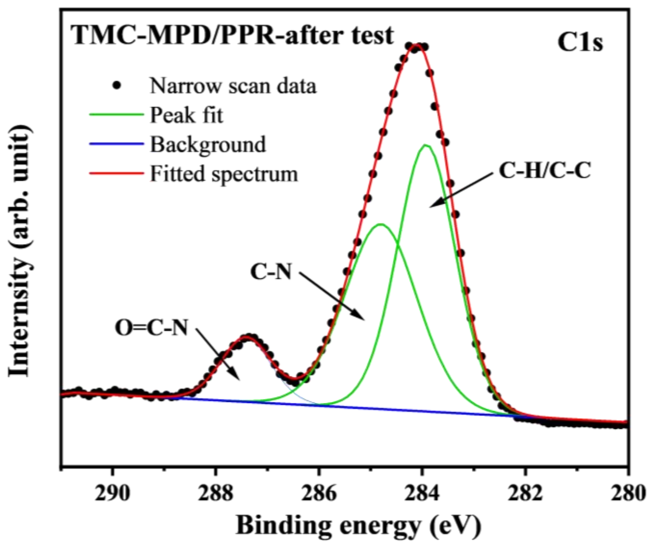

Figure S3 C1s XPS spectra of the TMC-MPD/PPR membrane after test

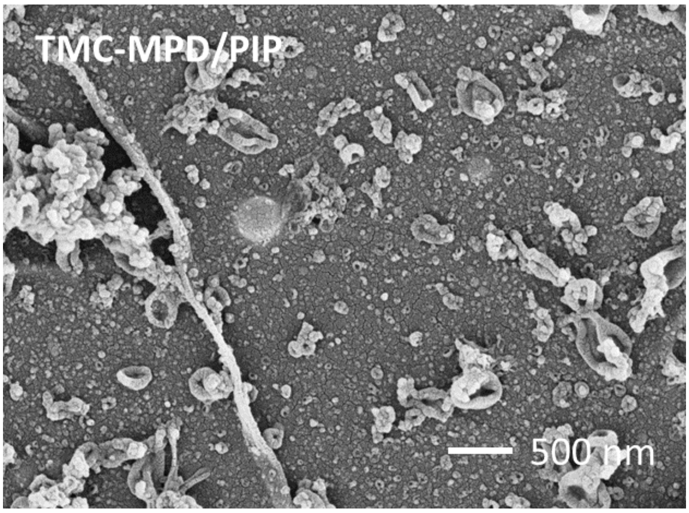

Figure S4 Surface SEM image of membrane fabricated by adding 10% PIP.
